# Robust Rate Maximization for Heterogeneous Wireless Networks under Channel Uncertainties

**DOI:** 10.3390/s18020639

**Published:** 2018-02-21

**Authors:** Yongjun Xu, Yuan Hu, Guoquan Li

**Affiliations:** 1 School of Communication and Information Engineering, Chongqing University of Posts and Telecommunications, Chongqing 400065, China; xuyj@cqupt.edu.cn (Y.X.), huycq1995@163.com (Y.H.); 2 State Key Laboratory of Integrated Services Networks, Xidian University, Xi’an 710071, China; xuyj12@126.com

**Keywords:** heterogeneous wireless networks, femtocell networks, channel uncertainties, robust resource allocation, quality of service

## Abstract

Heterogeneous wireless networks are a promising technology in next generation wireless communication networks, which has been shown to efficiently reduce the blind area of mobile communication and improve network coverage compared with the traditional wireless communication networks. In this paper, a robust power allocation problem for a two-tier heterogeneous wireless networks is formulated based on orthogonal frequency-division multiplexing technology. Under the consideration of imperfect channel state information (CSI), the robust sum-rate maximization problem is built while avoiding sever cross-tier interference to macrocell user and maintaining the minimum rate requirement of each femtocell user. To be practical, both of channel estimation errors from the femtocells to the macrocell and link uncertainties of each femtocell user are simultaneously considered in terms of outage probabilities of users. The optimization problem is analyzed under no CSI feedback with some cumulative distribution function and partial CSI with Gaussian distribution of channel estimation error. The robust optimization problem is converted into the convex optimization problem which is solved by using Lagrange dual theory and subgradient algorithm. Simulation results demonstrate the effectiveness of the proposed algorithm by the impact of channel uncertainties on the system performance.

## 1. Introduction

With the exponential growth of mobile data driven by various communication applications, such as WIFI and smartphones, the traditional wireless network via macrocell base stations (BSs) can not satisfy higher communication requirements (e.g., throughput and coverage). As a new candidate technology in the fifth generation (5G) wireless communication networks, heterogeneous network is proposed to improve network coverage and data rate [1,2,3]. In heterogeneous networks, many femtocells with low-cost and low-energy consumption are distributed around macro BSs randomly meanwhile femtocell users (FUs) share the same spectrum resource with macrocell users (MUs) to obtain better spectral efficiency (e.g., co-channel development) and supplement traditional single-tier cellular systems. However, the cross-tier interference from femtocell users to macrocell receivers should be controlled severely. Therefore, interference mitigation is very important for which power control-based resource allocation (i.e., power allocation) has been a feasible method for Heterogeneous wireless networks (HetNets) [4,5].

The design of resource allocation algorithms in HetNets has attracted much research interest owing to its importance. The main task in resource allocation of underlay femtocell networks is to reduce the interference power received at MUs and simultaneously obtain the expected performance of femtocells, such as maximize the throughput of femtocells. To achieve this objective, various resource allocation (i.e., power control) algorithms have been extensively studied for various scenarios in [6,7,8]. In [6], a resource allocation problem in both the uplink and the downlink for two-tier femtocell networks is considered for maximizing the capacity of sensitive FUs and delay-tolerant FUs under the cross-tier interference constraint of MU and the quality-of-service (QoS) constraint of delay-sensitive users. In [7], the authors propose an interference mitigation strategy to improve the uplink throughput by setting a fixed interference threshold and adjusting the maximum transmit power of femtocell user. To enhance the energy efficiency (EE) of HetNet, in [8], two EE resource allocation algorithms via game theory are proposed for downlink transmission in multichannel macro-femto networks. Note that all the literatures above are designed based on the assumption of perfect knowledge of channel state information (CSI) at the transmitters. Although they have generally been assumed that complete system information (i.e., perfect channel state information) are available to FUs, nevertheless, due to the random nature of wireless channels and inaccurate channel estimation, as well as channel delays, it is impossible for FUs to obtain exact values of system parameters, such as channel gains and interference power from other networks.

In fact, due to lack of cooperation between macrocell networks and femtocell networks, obtaining complete system information pertaining to MUs is difficult for FUs. For instance, uncertain channel gains between FUs and MUs’ receivers may cause the sum aggregated interference of FUs on the MUs’ receivers to exceed the tolerable threshold, which means that the QoS requirements of users in the macrocell can not be guaranteed. As a result, from the perspective of MUs, this would increase the outage probability of MUs. Additionally, uncertainties on channel gains between femtocell BS and FUs’ receivers may also reduce the minimum rate requirement or actual signal-to-interference-plus-noise-ratio (SINR) of each FU at its femtocell BS or receiver below the target threshold. Therefore, the robustness of resource allocation algorithms should be considered ahead of time.

Thanks to robust optimization theory [9,10], robust resource allocation algorithms under imperfect CSI have drawn considerable attentions to overcome the effect of uncertainties in HetNets. Generally, the robust resource allocation designs are developed by two types of CSI errors, i.e., worst-case model and stochastic model [11]. The deterministic model assumes that the instantaneous value of uncertain parameter is bounded by the worst-case upper boundary (e.g., ellipsoidal uncertainty sets), which aims to obtain the worst-case robustness and guarantee. On the contrary, the stochastic approach assumes that the statistical information of CSI or channel estimation errors is known at the transmitters and seeks to a suboptimal solution for maintaining a certain outage probability on average, such as probabilistic SINR constraint. In [12], with the consideration of ellipsoidal uncertainty sets, Vaezpour et al. propose a robust distributed resource allocation algorithm in orthogonal frequency division multiple access (OFDMA) based femtocell networks that maximizes the total rate of FUs under the constraints on the minimum data requirement of each FU and the co-tier as well as cross-tier interference. In [13], based on a robust Stackelberg game, a downlink power control scheme under column-wise uncertainty model is proposed for maximizing the capacities of each FU and each MU, where the impact of uncertainty and Nash equilibrium point are analyzed by using variational inequality (VI) and Stackelberg game theory respectively. In [14], taking the ellipsoidal channel uncertainties into account, Xiao et al. present a robust resource allocation algorithm in full-duplex based OFDMA femtocell HetNets considering the throughput maximization of the femtocell while avoiding harmful interference to the macrocell. In [15], based on game theory, a robust power control scheme for heterogeneous users is proposed by taking the bounded channel uncertainties into account. In [16], Xu et al. propose a robust resource allocation algorithm for multiuser HetNets with macrocells and microcells through geometrical programming method. However, in practice, the case of worst-case estimation error may not always exist so that the proposed algorithms sacrifice system performance in a large part.

Moreover, the upper bound of uncertainty can not be easily obtained in practical Heterogeneous systems. Nevertheless, the robust resource allocation problem with Bayesian approach (i.e., probability constraint [17]) is more appropriate for stochastic scenario because of the stochastic nature of measurements and estimation errors in wireless networks. In [18,19], based on the hierarchical game theory, Liu et al. propose a robust uplink power allocation algorithm to minimize the transmit power of FUs for two-tier femtocell networks sharing the same frequency with macrocells, where the uncertainties of channel gains are formulated as the probabilistic constraints. But these algorithms aim to minimize the power consumption, and thus cannot be used for the rate maximization in HetNets under channel uncertainties. In [20], Mokari et al. investigate the robust quantized ergodic resource allocation scheme to achieve the sum-rate maximization of femtocell network while guaranteeing the macro network interference requirements with any desired high probability. However, only channel gains of interference links subject to probability constraint are considered. They do not consider the uncertainty of signal links. In [21], for a two-tier HetNet, a robust MISO transmit power optimization problem is considered to design a minimum power downlink beamformer under the outage-based QoS constraints. However, all the aforementioned work only focuses on the protections of MUs in outage-based form (e.g., probabilistic cross-tier interference constraint of each MU). Under channel uncertainties, both the rate requirement of each FU and the performance protection of MUs should be considered simultaneously. Furthermore, the sensitivity analysis should be given to address the robustness of the scheme. The summary of the related work about resource allocation of HetNets is listed in Table 1.

In this paper, we consider the robust uplink resource allocation algorithm in a two-tier heterogeneous network under the stochastic model. We assume that there is a maco BS serving multiple MUs, and multiple femto BSs intending to serve their own FUs inside the macrocell network’s coverage. In our formulation, we maximize the sum data rate of all FUs under the outage probability constraints that the cross-tier interference of MU and the rate requirement of FU are considered. Our main contributions are summarized as follows:

- A multiuser resource allocation model for a two-tier HetNet is built. In this paper, we consider that the perfect CSI in both interference links (i.e., from the FUs to MUs) and signal links (i.e., the signal links of femtocell network) are not obtained while the channel uncertainties of the cross-tier interference constraints of MUs and the minimum rate constraint of each FU are formulated into the probability constraints. The existing works only consider the uncertainties in interference links from FUs to MUs, which ignores the effect of channel uncertainties from inter-tier links among FUs. Due to channel uncertainties, the original problem is non-convex and computationally intractable.
- Furthermore, the original non-convex optimization problem is transformed into two different problems under no CSI feedback and partial CSI. Unlike traditional Bernstein inequality approximation approach with high computational complexity, however, in this paper, both of outage-based interference and rate constraints are transformed into the closed-form approximations by some algebraic transformation. A sub-optimal algorithm is proposed to solve the robust mixed integer programming problem by using the Lagrange dual decomposition and the sub-gradient method.
- Thirdly, the computational complexity and sensitivity analysis (i.e., the impacts of uncertain parameters) of the proposed algorithm are given in this paper. Simulation results demonstrate the effectiveness of the proposed algorithm in terms of converge performance by comparing with the existing robust power control algorithm.

The rest of this paper is organized as follows. Section 2 introduces the system model. Section 3 gives the robust resource allocation problem. Section 4 gives the transformation of outage probability constraint and shows the robust power allocation algorithm. And, the performance analysis and Simulation results are provided in Section 5, and conclusions are drawn in Section 6.

## 2. System Model

**Figure 1:**
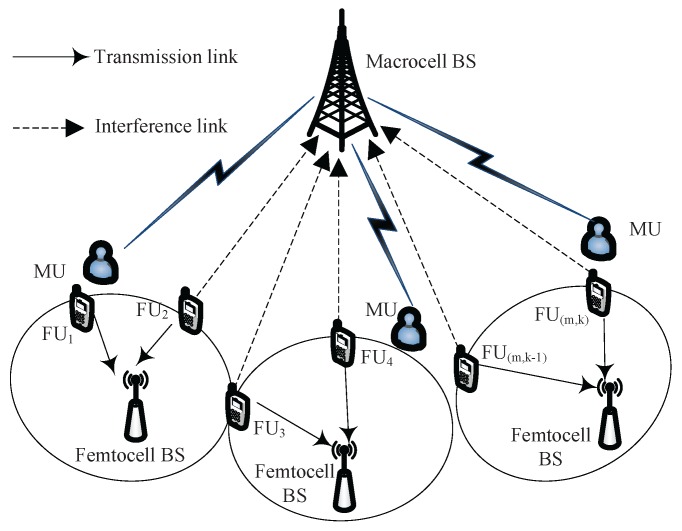
An example of the two-tier HetNet consisting of one macrocell and multiple femtocells.

We consider a multiuser OFDM uplink HetNet comprising *M* FUs communicating with their femtocell BSs over *K* subcarriers. The FUs can utilize the spectrum resource of MUs by femtocell BSs opportunistically, as shown in Figure 1. Note that both *M* and *K* vary based on the number of active users and available vacant subcarriers dynamically, and indexed by m∈M≜{1,2,⋯,M} and k∈K≜{1,2,⋯,K} respectively. Suppose that both of BSs and user equipments (UEs) have single antenna. Further, we assume K≥M, and the bandwidth of each subcarrier is assumed to be *B* Hz which is much less than the coherent bandwidth of wireless channel. As a result, users undergo a flat fading. The notations are summarized in Table 2.

According to the definition of Table 1, based on Shannon theorem, the corresponding data rate of FU *m* over subcarrier *k* is formulated as

(1) $$\begin{array}{c} r_{m,k}=B\rho_{m,k}\log_{2}\left(1+\frac{p_{m,k}h_{m,k}}{\sigma_{m,k}^{}}\right) \end{array}$$

where the $\rho_{m,k}$ denotes subcarrier assignment of FU *m* on subcarrier *k*, which can only be either 1 or 0, indicating whether the subcarrier *k* is used by the FU *m* or not.

Due to the limitation of battery capacity of the *m*th FU transmitter, the transmit power of each FU is not infinite, and the constraint is denoted by

(2) $$\begin{array}{c} \sum_{k=1}^{K}\rho_{m,k}p_{m,k}\leq p_{m}^{max},\forall m\in M \end{array}$$

where $p_{m}^{max}$ is the maximum transmit power of FU *m*.

Meanwhile, data rate should satisfy a minimum requirement to protect the QoS of FU *m*, given by

(3) $$\begin{array}{c} \sum_{k=1}^{K}r_{m,k}\geq R_{m}^{min},\forall m\in M \end{array}$$

where $R_{m}^{min}$ denotes minimum rate requirement of FU *m*.

The total cross-tier interference constraint from femtocell networks to MU receiver can be expressed as

(4) $$\begin{array}{c} \sum_{m=1}^{M}\sum_{k=1}^{K}\rho_{m,k}p_{m,k}g_{m,k}\leq I^{th} \end{array}$$

where $I^{th}$ is the interference temperature level at MU-receiver.

The sum rate maximization based Power Allocation (PA) problem for HetNets can be written as

(5) $$\begin{array}{c} \;\;\max_{\rho_{m,k},p_{m,k}}\;\sum_{m=1}^{M}\sum_{k=1}^{K}r_{m,k}\\ s.t.C_{1}:\;\sum_{k=1}^{K}\rho_{m,k}=1,\forall m\in M,\\ \;\;C_{2}:\;\sum_{k=1}^{K}\rho_{m,k}p_{m,k}\leq p_{m}^{max},\forall m\in M,\\ \;\;C_{3}:\;\sum_{k=1}^{K}r_{m,k}\geq R_{m}^{min},\forall m\in M,\\ \;\;C_{4}:\;\sum_{m=1}^{M}\sum_{k=1}^{K}\rho_{m,k}p_{m,k}g_{m,k}\leq I^{th},\\ \;\;C_{5}:\rho_{m,k}\in \left\{0,1\right\},\forall m\in M,\;k\in K. \end{array}$$

$C_{1}$ ensures that each subcarrier *k* is only assigned to one FU, where $\rho_{m,k}=1$ means that the *k*th subcarrier is used by FU *m*. Moreover, $C_{2}$ represents the transmission power constraint of FU *m* over subcarriers. $C_{3}$ can ensure the QoS of each FU. $C_{4}$ denotes the total interference power to the MU receiver. The problem with integer variable $\rho_{m,k}=1$ is a non-convex and mixed integer programming problem. Assume that the MU can provide the information of channel gains $g_{m,k}$ feedback to the FU, which means that channel gains can be accurately estimated by FUs. Most of current power allocation in HetNets focus on optimal power control under perfect CSI [22,23].

In practice, however, channel uncertainties are inevitable due to estimation errors and quantization errors which cause harmful interference to the MUs [24]. To reduce performance degradation of MUs, PA with channel uncertainties should be considered in advance. However, to the best of our knowledge, robust PA considering the QoS of both FUs and MUs has not been investigated in the existing works.

## 3. Robust Power Allocation Problem

Due to lack of cooperation between femtocell network and macrocell network, it is a difficult task to precisely estimate the channel gain $g_{m,k}$ from FU-transmitter to MU-receiver. In this paper, our goal is to design a robust PA and subcarrier assignment scheme to ensure the communication quality of FUs and MUs under channel uncertainties. Hence, we reformulated the constraints $C_{3}$ and $C_{4}$ as probability form. The PA Problem (5) with outage probability constraints is formulated as

(6) $$\begin{array}{c} \;\;\max_{\rho_{m,k},p_{m,k}}\;\sum_{m=1}^{M}\sum_{k=1}^{K}r_{m,k}\\ s.t.\;C_{1},\;C_{2},\;C_{5},\\ \;\;C_{6}:\;\mathrm{Pr}\left\{\sum_{k=1}^{K}r_{m,k}<R_{m}^{min}\right\}\leq \xi_{m},\forall m\in M,\\ \;\;C_{7}:\;\mathrm{Pr}\left\{\sum_{m=1}^{M}\sum_{k=1}^{K}\rho_{m,k}p_{m,k}g_{m,k}>I^{th}\right\}\leq \varepsilon . \end{array}$$

where both of $C_{6}$ and $C_{7}$ ensure that the QoS of MU and FU are satisfied with a certain probability of outage event that is less than the outage probability threshold $\xi_{m}$ and ε, respectively. In general, Problem (6) is a challenging optimization problem for obtaining analytical solutions because probabilistic rate and interference constraints (i.e., $C_{6}$ and $C_{7}$) are intractable and are not convex. The key step in the development of a tractable robust PA algorithm is to transform the outage probability constraints into efficiently-computable representation (e.g., convex form).

In the following part, we will transform the outage probability constraints into the deterministic and convex ones under imperfect statistical distribution model of uncertain parameter and perfect probability distributions of channel estimation errors.

### 3.1. Transformation without Statistical Information

In this section, the probabilistic interference constraint and probabilistic rate constraint are converted into the deterministic ones.

Due to the feature of OFDM technology, there is no mutual interference among different subcarriers, so that the data of each FU is mutually independent for all subcarriers. Define the rate set

(7) $$R^{k}=\left\{r_{m,k}\leq R_{m}^{min}\right\},$$

and

(8) $$\bar{R}=\left\{\sum_{k=1}^{K}r_{m,k}\leq R_{m}^{min}\right\}.$$

According to the above definition, the set $\bar{R}$ is a subset of the intersection of $R^{k}$, i.e.,

(9) $$\bar{R}\subset R=R^{1}\cap R^{2}\cap \cdots R^{K}.$$

According to probability theory, we have the following relationship,

(10) $$\mathrm{Pr}\left\{\bar{R}\right\}\leq \mathrm{Pr}\left\{R\right\}=\prod_{k=1}^{K}\mathrm{Pr}\left\{R^{k}\right\}.$$

In other words, we have

(11) $$\mathrm{Pr}\left\{\sum_{k=1}^{K}r_{m,k}\leq R_{m}^{min}\right\}\leq \prod_{k=1}^{K}\mathrm{Pr}\left\{r_{m,k}\leq R_{m}^{min}\right\}.$$

If the upper bound of probabilistic rate constraint satisfies the outage probability requirement, based on the worst-case method, as we know, the stochastic constraint $C_{6}$ is satisfied. Therefore, we have

(12) $$\max\mathrm{Pr}\left\{\sum_{k=1}^{K}r_{m,k}\leq R_{m}^{min}\right\}=\prod_{k=1}^{K}\mathrm{Pr}\left\{r_{m,k}\leq R_{m}^{min}\right\}\leq \xi_{m},$$

From (12), we have the deterministic form of outage-based probability constraint $C_{6}$, i.e.,

(13) $$R_{m}^{min}\leq B\rho_{m,k}\log_{2}\left(1+\frac{p_{m,k}}{\sigma_{m,k}}H_{h_{m,k}}^{-1}\left(\xi_{m}/K\right)\right),\forall m\in M.$$

If transmission power of SU satisfies the constraint (13), the outage probability of $C_{6}$ can be ensured. The proof of (13) is given in Appendix A.

Similarly, the probabilistic interference constraint $C_{7}$ can be transformed as

(14) $$\rho_{m,k}p_{m,k}\leq \frac{I^{th}}{KG_{g_{m,k}}^{-1}\left(\sqrt[{MK}]{1-\varepsilon }\right)},\forall m\in M,\forall k\in K.$$

Therefore, the outage probability constraint $C_{7}$ becomes a deterministic one. To keep the presentation concise, the proof of (14) is given in Appendix B.

Based on (13) and (14), the PA problem without knowledge of statistical model is given as

(15) $$\begin{array}{c} \;\;\max_{\rho_{m,k},p_{m,k}}\;\sum_{m=1}^{M}\sum_{k=1}^{K}r_{m,k}\\ s.t.\;C_{1},\;C_{2},\;C_{5},\\ C_{8}:B\rho_{m,k}\log_{2}\left(1+\frac{p_{m,k}}{\sigma_{m,k}}H_{h_{m,k}}^{-1}\left(\xi_{m}/K\right)\right)\geq R_{m}^{min},m\in M\\ C_{9}:K\rho_{m,k}p_{m,k}G_{g_{m,k}}^{-1}\left(\sqrt[{MK}]{1-\varepsilon }\right)\leq I^{th}. \end{array}$$

Obviously, problem (6) becomes a deterministic one. If the inverse cumulative distribution functions (CDFs) of variables (e.g., $g_{m,k}$ and $h_{m,k}$) are well-known (i.e., $G_{g_{m,k}}^{-1}\left(.\right)$ and $H_{h_{m,k}}^{-1}\left(.\right)$), the problem (15) can be easily solved. For example, if the channel gains are follow Rayleigh fading models, the problem can be well solved. In real communication scenario, however, due to the mobility of UEs, it is impractical to assume same fading models during different environments. Therefore, it is necessary to find a general method to solve this problem.

### 3.2. Transformation with Perfect Statistical Model

In order to address the above problem, it is feasible to find the solution with knowledge of perturbation part of uncertain channel gains.

If there are some errors in the estimated channel gain from feedback quantization or channel estimation [25]. Channel uncertainties can be formulated as an additive model of uncertain parameter [11], i.e.,

(16) $$\left\{\begin{array}{c} g_{m,k}={\hat{g}}_{m,k}+\Delta g_{m,k},\forall m\in M,\forall k\in K,\\ h_{m,k}={\hat{h}}_{m,k}+\Delta h_{m,k},\forall m\in M,\forall k\in K, \end{array}\right.$$

where ${\hat{g}}_{m,k}$ is the estimated channel gain between FU-transmitter and MU-receiver. And ${\hat{h}}_{m,k}$ denotes the estimated channel gains from FUs to their BS. Those parameters are perfectly known for FUs. $\Delta g_{m,k}$ and $\Delta h_{m,k}$ are the corresponding perturbation terms (i.e., estimation error).

It is clear that FUs can obtain the CSI by estimating the channels between FUs and MUs so that the causes of CSI errors are from channel estimation, as a result, this type of estimation error can be formulated as independent Gaussian distribution model [25]. Therefore, uncertain parameter $\Delta g_{m,k}$ is reasonably assumed to be distributed random with zero-mean and variance $\upsilon_{m,k}^{2}$, i.e., $\Delta g_{m,k}∼CN\left(0,\upsilon_{m,k}^{2}\right)$. However, the elements $\Delta h_{m,k}$ in (16) should be assumed to follow the independent uniform distributed random variables, i.e., $\Delta h_{m,k}\in \left[-\delta_{m,k},\delta_{m,k}\right]$, where $\delta_{m,k}$ denotes the upper bound of uncertain region. The reason is that the uplink channel gain from FU’s transmitter to the BS is obtained by a quantizer to quantize CSI and feed it back to its corresponding FU’s transmitter.

To protect the performance of MUs, each FU transmits less power for avoiding harmful interference to the MU over link *k*. Based on $C_{6}$ and Model (16), we have

(17) $$\begin{array}{c} \mathrm{Pr}\left\{\sum_{k=1}^{K}B\rho_{m,k}\log_{2}\left(1+\frac{p_{m,k}\left({\hat{h}}_{m,k}+\Delta h_{m,k}\right)}{\sigma_{m,k}}\right)<R_{m}^{min}\right\}\leq \mathrm{Pr}\left\{\left|C_{m}\right|B\rho_{m,k}\log_{2}\left(1+\frac{p_{m,k}\left({\hat{h}}_{m,k}+\Delta h_{m,k}\right)}{\sigma_{m,k}}\right)\leq R_{m}^{min}\right\}\\ \;=\mathrm{Pr}\left\{\Delta h_{m,k}\leq D_{m,k}\right\}\leq \xi_{m}, \end{array}$$

where $C_{m}$ denotes the subcarrier set of FU *m*, and $\left|C_{m}\right|$ represents the number of subcarrier that is chosen by FU *m*. And $D_{m,k}=\frac{\sigma_{m,k}}{p_{m,k}}\left(2^{R_{m}^{min}\;/\;\left|C_{m}\right|B\rho_{m,k}}-1\right)-{\hat{h}}_{m,k}$.

Due to uniform distribution of channel errors $\Delta h_{m,k}$, we have

(18) $$2^{R_{m}^{min}\;/\;\left|C_{m}\right|B\rho_{m,k}\leq 1+\frac{p_{m,k}}{\sigma_{m,k}}\left({\hat{h}}_{m,k}+2\delta_{m,k}\xi_{m}\right),}$$

and

(19) $$\sum_{k\in C_{m}}^{}B\rho_{m,k}\log_{2}\left(1+\frac{p_{m,k}}{\sigma_{m,k}}\left({\hat{h}}_{m,k}+2\delta_{m,k}\xi_{m}\right)\right)\geq R_{m}^{min}.$$

As a result, the probabilistic rate constraint becomes a deterministic one as form of (19).

According to the relationship of (16) and constraint $C_{7}$, we have

(20) $$\mathrm{Pr}\left\{\sum_{m=1}^{M}\sum_{k=1}^{K}\rho_{m,k}p_{m,k}\Delta g_{m,k}>\bar{I}\right\}\leq \varepsilon ,$$

where $\bar{I}=I^{th}-\sum_{m=1}^{M}\sum_{k=1}^{K}\rho_{m,k}p_{m,k}{\hat{g}}_{m,k}$ is a deterministic term for FU’s transmitter. The Constraint (20) can also be reformulated as

(21) $$\mathrm{Pr}\left\{\sum_{m=1}^{M}\sum_{k\in C_{m}}\rho_{m,k}p_{m,k}\Delta g_{m,k}\leq \bar{I}\right\}\geq 1-\varepsilon .$$

To satisfy the outage probability requirement, the interference constraint of left side in (21) must hold under any channel estimation error.

Based on worst-case principle, we have

(22) $$\begin{array}{cc} \sum_{m=1}^{M}\sum_{k\in C_{m}}\rho_{m,k}p_{m,k}\Delta g_{m,k} & \leq \max_{\Delta g_{m,k}\in R_{g}}\left(\sum_{m=1}^{M}\sum_{k\in C_{m}}\rho_{m,k}p_{m,k}\Delta g_{m,k}\right)\\ & \leq \sum_{m=1}^{M}\left|C_{m}\right|\rho_{m,k}p_{m,k}\Delta g_{m,k^{\prime }}, \end{array}$$

where $k^{\prime }=\arg\max_{k}\left({\hat{g}}_{m,k}\right)$ denotes the worst interference link between FU and MU. $\left|\cdot \right|$ denotes the number of element in set.

In other words, if the outage-based constraint can be ensured under the case of worst errors over all subcarriers, the outage performance of MU must be kept. Consequently, we have

(23) $$\mathrm{Pr}\left\{\sum_{m=1}^{M}\left|C_{m}\right|\rho_{m,k}p_{m,k}\Delta g_{m,k^{\prime }}\leq \bar{I}\right\}\geq 1-\varepsilon .$$

Define $B_{m}=\left|C_{m}\right|\rho_{m,k}p_{m,k}$ and ${\tilde{B}}_{m}=B_{m}\Delta g_{m,k^{\prime }}$. Since there is $\Delta g_{m,k^{\prime }}∼CN\left(0,\upsilon_{m,k^{\prime }}^{2}\right)$, the variable ${\tilde{B}}_{m}$ follows the Gaussian distribution with zero mean and variance $B_{m}^{2}\upsilon_{m,k^{\prime }}^{2}$, i.e., ${\tilde{B}}_{m}∼CN\left(0,{\left(B_{m}\upsilon_{m,k^{\prime }}\right)}^{2}\right)$. As a result, we have

(24) $$\begin{array}{cc} \mathrm{Pr}\left\{\sum_{m=1}^{M}\left|C_{m}\right|\rho_{m,k}p_{m,k}\Delta g_{m,k^{\prime }}\leq \bar{I}\right\} & =\mathrm{Pr}\left\{\sum_{m=1}^{M}B_{m}\Delta g_{m,k^{\prime }}\leq \bar{I}\right\}\\ & =\mathrm{Pr}\left\{\sum_{m=1}^{M}{\tilde{B}}_{m}\leq \bar{I}\right\}\geq 1-\varepsilon . \end{array}$$

Since the sum of Gaussian random variable ${\tilde{B}}_{m}$ is still obeying Gaussian distribution, i.e., $\hat{B}=\sum_{m=1}^{M}{\tilde{B}}_{m}∼CN\left(0,\sigma^{2}\right)$ and $\sigma =\sqrt{\sum_{m=1}^{M}{\left(B_{m}\upsilon_{m,k^{\prime }}\right)}^{2}}$, therefore, the expression of (24) can be reformulated as

(25) $$\mathrm{Pr}\left\{\hat{B}\leq \bar{I}\right\}=Q\left(\frac{\bar{I}-0}{\sigma }\right)\geq 1-\varepsilon ,$$

where Q(x) is a Gaussian Q-function with the expression of $Q\left(x\right)=\frac{1}{\pi }\int_{0}^{\pi \;/\;2}\exp(-\frac{x^{2}}{2\sin^{2}\theta })d\theta$. As a result, we have

(26) $$\begin{array}{c} \bar{I}\geq Q^{-1}\left(1-\varepsilon \right)\sqrt{\sum_{m=1}^{M}{\left(B_{m}\upsilon_{m,k^{\prime }}\right)}^{2}}, \end{array}$$

where $Q^{-1}\left(\cdot \right)$ denotes the inverse Gaussian Q-function. Combining with the inequality relationship $\sqrt{\sum_{i}x_{i}^{2}}\leq \sum_{i}\sqrt{x_{i}^{2}}$, we can use the upper bound of right side in (26) to obtain the decomposable form, i.e.,

(27) $$\sqrt{\sum_{m=1}^{M}{\left(B_{m}\upsilon_{m,k^{\prime }}\right)}^{2}}\leq \sum_{m=1}^{M}{B_{m}\upsilon_{m,k^{\prime }}}^{2}$$

As a result, the outage-based probability constraint *C*_6_ becomes a deterministic form

(28) $$\sum_{m=1}^{M}\sum_{k\in C_{m}}^{}\rho_{m,k}p_{m,k}\left({\hat{g}}_{m,k}+Q^{-1}\left(1-\varepsilon \right)\upsilon_{m,k^{\prime }}\right)\leq I^{th}.$$

Define ${\hat{r}}_{m,k}=B\rho_{m,k}\log_{2}\left(1+\frac{p_{m,k}}{\sigma_{m,k}}\left({\hat{h}}_{m,k}+2\delta_{m,k}\xi_{m}\right)\right)$, combining (6), (19) and (28), we obtain the considerable robust resource allocation problem as follows

(29) $$\begin{array}{c} \;\;\max_{\rho_{m,k},p_{m,k}}\;\sum_{m=1}^{M}\sum_{k\in C_{m}}{\hat{r}}_{m,k}\\ s.t.\;C_{1},C_{2},C_{5},\\ \;\;C_{10}:\sum_{m=1}^{M}\sum_{k\in C_{m}}\rho_{m,k}p_{m,k}\left({\hat{g}}_{m,k}+Q^{-1}\left(1-\varepsilon \right)\upsilon_{m,k^{\prime }}\right)\leq I^{th},\\ \;\;C_{11}:\sum_{k\in C_{m}}{\hat{r}}_{m,k}\geq R_{m}^{min},\forall m\in M. \end{array}$$

As a result, the probabilistic optimization problem (6) is transformed into a non-probabilistic one (29).

## 4. Robust Resource Allocation Algorithm

Obviously, Problem (29) is still not a convex problem due to the integer variable $\rho_{m,k}$. Since both the real variable $p_{m,k}$ and integer variable $\rho_{m,k}$ are in the optimization problem, it conducts a mixed integer programming problem. Thus, we relax the subcarrier assignment factor into continuous one and introduce a variable $s_{m,k}=\rho_{m,k}p_{m,k}$ for any FU and subcarrier, where $\rho_{m,k}\in \left[0,1\right]$ indicates a time-division multiple access (TDMA) strategy for different FUs. Therefore, the Problem (29) becomes a convex optimization problem as follows,

(30) $$\begin{array}{c} \;\;\max_{\rho_{m,k},s_{m,k}}\;\sum_{m=1}^{M}\sum_{k\in C_{m}}{\tilde{r}}_{m,k}\\ s.t.\;C_{1},\\ \;\;C_{12}:\sum_{k\in C_{m}}s_{m,k}\leq p_{m}^{max},\forall m\in M,\\ \;\;C_{13}:\;\sum_{k\in C_{m}}{\tilde{r}}_{m,k}\geq R_{m}^{min},\forall m\in M,\\ \;\;C_{14}:\;\sum_{m=1}^{M}\sum_{k\in C_{m}}s_{m,k}\left({\hat{g}}_{m,k}+Q^{-1}\left(1-\varepsilon \right)\upsilon_{m,k}\right)\leq I^{th}, \end{array}$$

where ${\tilde{r}}_{m,k}=B\rho_{m,k}\log_{2}\left(1+\frac{s_{m,k}\left({\hat{h}}_{m,k}+2\delta_{m,k}\xi_{m}\right)}{\sigma_{m,k}\rho_{m,k}}\right)$ denotes the effective data rate due to unknown channel gain $h_{m,k}$ (i.e., true physical channel gain). The objective function in (30) is concave since the corresponding hessian matrix can be proved to be negative semi-definiteness by using the Lemma 1 in [26]. In addition, the constraints are linear so that the robust optimization Problem (30) is a convex problem.

We can obtain the analytical solutions by using Lagrangian methods [27]. To deal with the optimization problem (30), we first define a Lagrangian function as

(31) $$\begin{array}{c} L\left(\left\{s_{m,k}\right\},\left\{\rho_{m,k}\right\},\left\{\lambda_{m}\right\},\left\{\mu_{m}\right\},\left\{\beta_{m}\right\},\upsilon \right)=\sum_{m=1}^{M}\sum_{k\in C_{m}}{\tilde{r}}_{m,k}-\sum_{m=1}^{M}\lambda_{m}\left(\sum_{k\in C_{m}}s_{m,k}-p_{m}^{max}\right)\\ -\sum_{m=1}^{M}\beta_{m}\left(\sum_{k\in C_{m}}\rho_{m,k}-1\right)-\upsilon \left(\sum_{m=1}^{M}\sum_{k\in C_{m}}s_{m,k}\left({\hat{g}}_{m,k}+Q^{-1}\left(1-\varepsilon \right)\upsilon_{m,k}\right)-I^{th}\right)\\ -\sum_{m=1}^{M}\mu_{m}\left(R_{m}^{min}-\sum_{k\in C_{m}}{\tilde{r}}_{m,k}\right), \end{array}$$

where $\left\{\lambda_{m}\right\}$, $\left\{\mu_{m}\right\}$, $\left\{\beta_{m}\right\}$ and υ are the nonnegative Lagrangian multipliers for the corresponding constraints in (30), respectively. The Lagrangian dual function can be defined as

(32) $$\begin{array}{c} g\left(\left\{\lambda_{m}\right\},\left\{\beta_{m}\right\},\left\{\mu_{m}\right\},\upsilon \right)=\max_{\left\{s_{m,k}\right\},\left\{\rho_{m,k}\right\}}\;L\left(\left\{s_{m,k}\right\},\left\{\rho_{m,k}\right\},\left\{\lambda_{m}\right\},\left\{\beta_{m}\right\},\left\{\mu_{m}\right\},\upsilon \right). \end{array}$$

And the dual problem is formulated as

(33) $$\begin{array}{c} \min_{\left\{\lambda_{m},\beta_{m},\mu_{m},\upsilon \right\}}\;g\left(\left\{\lambda_{m}\right\},\left\{\beta_{m}\right\},\left\{\mu_{m}\right\},\upsilon \right)\\ s.t.\;\lambda_{m}\geq 0,\beta_{m}\geq 0,\mu_{m}\geq 0,\upsilon \geq 0. \end{array}$$

The Lagrangian dual Problems (32) and (33) can be decomposed into a master problem and M×K subproblems [27]. As a result, the Lagrangian function can be rewritten as

(34) $$\begin{array}{cc} L\left(\left\{s_{m,k}\right\},\left\{\rho_{m,k}\right\},\left\{\lambda_{m}\right\},\left\{\beta_{m}\right\},\left\{\mu_{m}\right\},\upsilon \right) & =\sum_{m=1}^{M}\sum_{k\in C_{m}}L_{m,k}\left(\left\{s_{m,k}\right\},\left\{\rho_{m,k}\right\},\left\{\lambda_{m}\right\},\left\{\beta_{m}\right\},\left\{\mu_{m}\right\},\upsilon \right)\\ & +\sum_{m=1}^{M}\lambda_{m}p_{m}^{max}-\sum_{m=1}^{M}R_{m}^{min}\mu_{m}+\sum_{m=1}^{M}\beta_{m}+I^{th}\upsilon , \end{array}$$

where

(35) $$\begin{array}{cc} L_{m,k}\left(\left\{s_{m,k}\right\},\left\{\rho_{m,k}\right\},\left\{\lambda_{m}\right\},\left\{\beta_{m}\right\},\left\{\mu_{m}\right\},\upsilon \right) & =\left(1+\mu_{m}\right){\tilde{r}}_{m,k}-\lambda_{m}s_{m,k}-\beta_{m}\rho_{m,k}\\ & -\upsilon s_{m,k}\left({\hat{g}}_{m,k}+Q^{-1}\left(1-\varepsilon \right)\upsilon_{m,k}\right). \end{array}$$

The Karush-Kuhn-Tucker (KKT) conditions of variables $s_{m,k}$ and $\rho_{m,k}$ can be obtained by calculating the derivatives of (35) with the optimal variables, i.e.,

(36) $$0\leq s_{m,k}⊥\frac{\partial L_{m,k}\left(\cdots \right)}{\partial s_{m,k}}\geq 0,$$

(37) $$0\leq \rho_{m,k}⊥\frac{\partial L_{m,k}\left(\cdots \right)}{\partial \rho_{m,k}}\geq 0,$$

where ⊥ denotes the orthogonality relationship of the corresponding variables. Additionally, the derivatives $\frac{\partial L_{m,k}\left(\cdots \right)}{\partial s_{m,k}}$ and $\frac{\partial L_{m,k}\left(\cdots \right)}{\partial \rho_{m,k}}$ are given as

(38) $$\begin{array}{c} \frac{\partial L_{m,k}\left(\cdots \right)}{\partial s_{m,k}}=\left(1+\mu_{m}\right)\frac{\partial {\tilde{r}}_{m,k}\left(s_{m,k},\rho_{m,k}\right)}{\partial s_{m,k}}-\lambda_{m}-\upsilon \left({\hat{g}}_{m,k}+Q^{-1}\left(1-\varepsilon \right)\upsilon_{m,k}\right), \end{array}$$

(39) $$\frac{\partial L_{m,k}\left(\cdots \right)}{\partial \rho_{m,k}}=\left(1+\mu_{m}\right)\frac{\partial {\tilde{r}}_{m,k}\left(s_{m^{*},k},\rho_{m,k}\right)}{\partial \rho_{m,k}}-\beta_{m}.$$

As a result, according to KKT conditions, we obtain the optimal transmit power

(40) $$\begin{array}{c} p_{m,k}^{*}=\frac{s_{m,k}}{\rho_{m,k}}={\left[\frac{1}{\ln2}\frac{B(1+\mu_{m})}{\lambda_{m}+\upsilon \left(\hat{g}+Q^{-1}\left(1-\varepsilon \right)\upsilon_{m,k}\right)}-\frac{\sigma_{m,k}}{{\hat{h}}_{m,k}+2\delta_{m,k}\xi_{m}}\right]}^{+}, \end{array}$$

where ${\left[x\right]}^{+}=\max\left(0,x\right)$. Moreover, the subcarrier *k* is assigned to the optimal user $m^{*}$, i.e.,

(41) $$\rho_{m,k}^{*}=1\left|{}_{m^{*}=\max_{m}\;{\hat{\beta }}_{m,k}}\right.,$$

where

(42) $$\begin{array}{c} {\hat{\beta }}_{m,k}=\left(1+\mu_{m}\right)\frac{\partial {\tilde{r}}_{m,k}\left(s_{m^{*},k},\rho_{m,k}\right)}{\partial \rho_{m,k}}=B\left(1+\mu_{m}\right)\left(\log_{2}\left(1+\frac{p_{m,k}^{*}{\hat{h}}_{m,k}}{\sigma_{m,k}}\right)\right.\left.-\frac{p_{m,k}^{*}{\hat{h}}_{m,k}}{\ln2\left(\sigma_{m,k}+p_{m,k}^{*}{\hat{h}}_{m,k}\right)}\right). \end{array}$$

**Figure 2:**
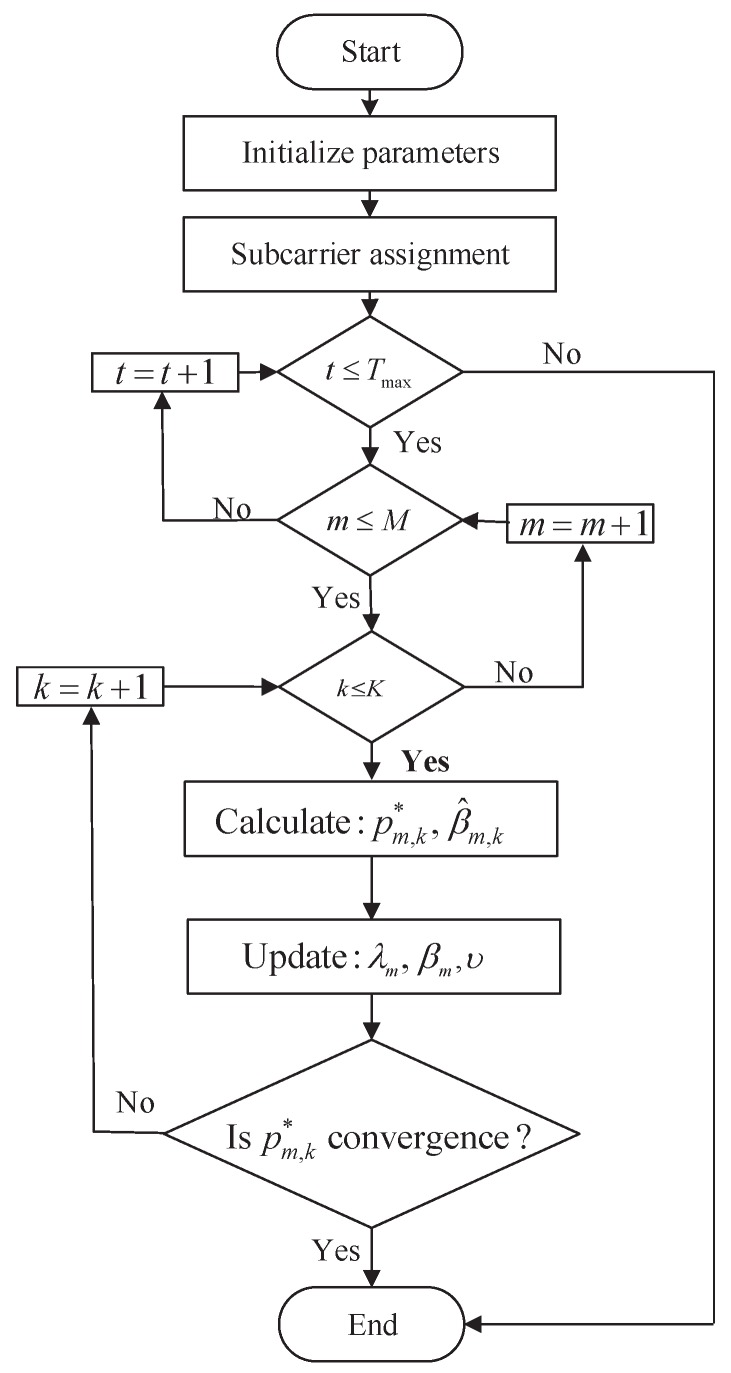
Algorithm flow chart.

The Lagrangian multipliers can be updated as follows

(43) $$\lambda_{m}^{t+1}={\left[\lambda_{m}^{t}-d_{1}^{t}\left(p_{m}^{max}-\sum_{k\in C_{m}}p_{m,k}^{t}\right)\right]}^{+},$$

(44) $$\mu_{m}^{t+1}={\left[\mu_{m}^{t}-d_{2}^{t}\left(\sum_{k\in C_{m}}{\tilde{r}}_{m,k}^{t}-R_{m}^{min}\right)\right]}^{+},$$

(45) $$\begin{array}{c} \upsilon^{t+1}=\left[\upsilon^{t}-d_{3}^{t}\left(I^{th}\right.\right.-{\left.\left.\sum_{m=1}^{M}\sum_{k\in C_{m}}p_{m,k}^{t}\left({\hat{g}}_{m,k}+Q^{-1}\left(1-\varepsilon \right)\upsilon_{m,k}\right)\right)\right]}^{+}. \end{array}$$

where *t* denotes the iteration number. $d_{1}$, $d_{2}$ and $d_{3}$ are the corresponding small step sizes. When the step sizes are sufficiently small, Lagrange multipliers can converge to equilibrium points [28]. The implementation procedure of the algorithm is given in Algorithm 1 and the algorithm flow chart is shown in Figure 2.

**Algorithm 1:** The implementation procedure.
1:Initialize maximum iteration number $T_{max}$; Set: iterations t=0, M>0, K>0; Lagrangian multipliers $\lambda_{m}\left(0\right)>0$, $\mu_{m}\left(0\right)>0$, and υ(0)>0; Outage probability thresholds ε∈(0,1), $\xi_{m}\in \left(0,1\right)$; Upper bound of channel estimation error in FU’s link is $\delta_{m,k}\in \left[0,1\right]$; Variance of estimation error in FU-to-MU link is $\upsilon_{m,k}\in \left[0,1\right]$;2:Set maximum transmit power $p_{m}^{max}>0$ and initialize power $p_{m,k}>0$ with same initialization values among all subcarriers. Define interference $I^{th}$, randomly generate ${\hat{g}}_{m,k}$ and ${\hat{h}}_{m,k}$.3:Initialize $\rho_{m,k}$ with subcarrier assignment method in [29], group subcarrier set $C_{m}$ and calculate $\left|C_{m}\right|$.4:**repeat**5:**for** t=1 to $T_{max}$ **do**6: **for** m=1 to *M* **do**7:  **for** k=1 to *K* **do**8:   Calculate transmit power $p_{m,k}^{*}$ according to (40)9:   Calculate ${\hat{\beta }}_{m,k}$ by (42)10:   Calculate $\rho_{m,k}^{*}$ by using (41)11:   Calculate Lagrange multipliers $\lambda_{m}$, $\mu_{m}$, and υ from (43–45)12:  **end for**13: **end for**14: t=t+115:**end for**16:**until** $t=T_{max}$ or transmit power convergence


## 5. Performance Analysis

In this section, we will analyze the complexity, sensitivity and simulation results of the proposed algorithm.

### 5.1. Complexity Analysis

The the computational complexity of the proposed algorithm is analyzed in this subsection. Obviously, the calculation of (38) for each femtocell user on each subcarrier in each femtocell *k* implies MK operations, so that the worst-case complexity of search (37) (i.e., find the optimal $\rho_{m,k}^{*}$) needs MK operations in each iteration. If the subgradient methods used in (39)–(41) require $t_{m}$ iterations to converage, then the updates of Lagrange multipliers $\lambda_{m}$ and $\mu_{m}$ for ∀m,k need $O\left(MK\right)$ operations [30], and the computation of υ needs $O\left(1\right)$, so that the $t_{m}$ is a polynomial function of $M^{2}K^{2}$. Thus the total complexity of the algorithm is $O\left(M^{2}K^{2}t_{m}\right)$ [27]. Additionally, $t_{m}$ can be small enough when the initial values of Lagrange multipliers $\lambda_{m}\left(0\right)$, $\mu_{m}\left(0\right)$ and υ(0) are well chosen [6,22].

### 5.2. Sensitivity Analysis

In this section, we will give the exact perturbation version of sum rate gap under uncertainties $\Delta g_{m,k}$ and $\Delta h_{m,k}$. The reduction in the sum transmission rate of FUs can be formulated as

(46) $$\begin{array}{c} U_{gap}=-\sum_{m=1}^{M}\sum_{k\in C_{m}}\left(2\mu_{m}^{*}\delta_{m,k}\xi_{m}+\upsilon^{*}Q^{-1}\left(1-\varepsilon \right)\upsilon_{m,k}\right), \end{array}$$

where $\mu_{m}^{*}$ and $\upsilon^{*}$ denotes optimal Lagrangian multipliers. The proof is given in Appendix C.

### 5.3. Simulation Results

In this subsection, we use computer simulations to demonstrate the performance of the proposed algorithm in different scenarios. The simulation parameters are given in Table 3.

**Figure 3:**
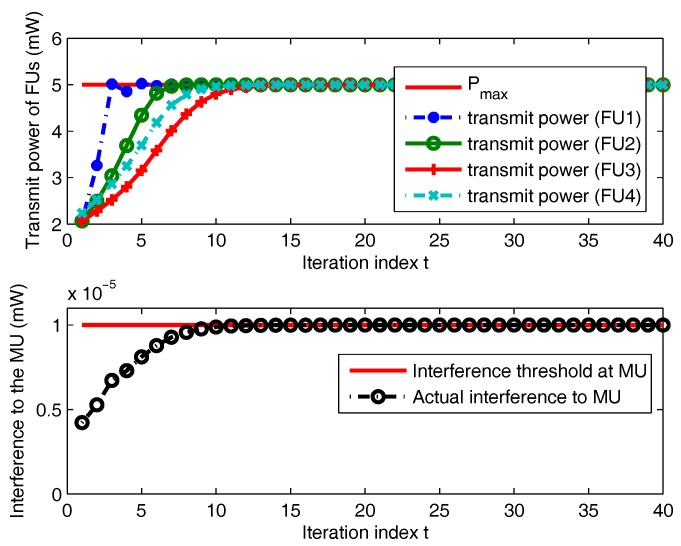
Convergence performance of the proposed algorithm.

Figure 3 shows the convergence performance of the proposed algorithm in terms of transmit power and interference power versus the number of iterations *t*. We can see that the proposed algorithm takes only small iterations to converge, which indicates that it has a good real-time performance for practical applications. The transmit power of each FU is constrained by the maximum transmit power level. In addition, our proposed algorithm can well protect the performance of MU because the corresponding interference power does not exceed the interference temperature threshold.

**Figure 4:**
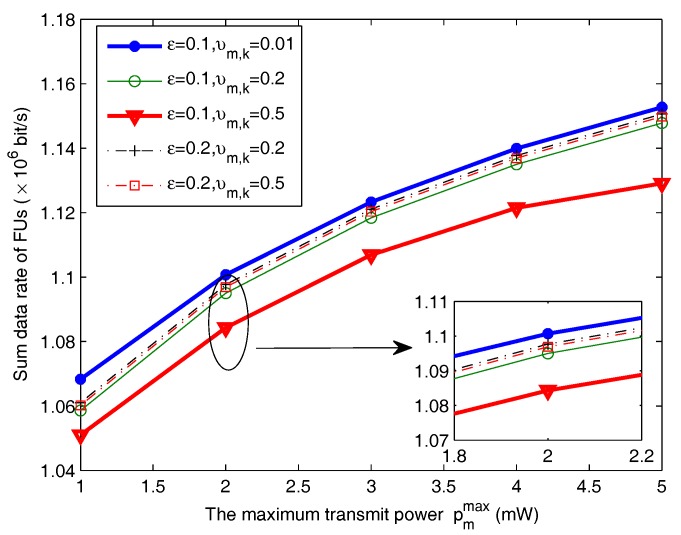
Sum data rate of FUs versus the maximum transmit power under different MU outage probabilities.

In Figure 4, the transmission rate of FUs is depicted as a function of ε under different channel estimation errors $\Delta g_{m,k}$ (i.e., the corresponding variance $\upsilon_{m,k}$). The outage probability of FU is defined as $\xi_{m}=0.1$. The variance of channel estimation error $\Delta h_{m,k}$ is $\delta_{m,k}=0.01$. As can be seen, the total data rate of FUs increases as the maximum transmit power threshold $p_{m}^{max}$ increases. This can be explained, as for lower values of $p_{m}^{max}$, the total transmit power of FUs $p_{m,k},\forall k$ is limited. Increasing the transmit power threshold $p_{m}^{max}$ can increase the feasible region of transmit power $p_{m,k}$. Hence, it enables the proposed algorithm to improve the sum data of FUs. In addition, the total data rate of FUs under the higher outage threshold of MU ε is higher than that of lower outage probability, e.g., rate$\left(\varepsilon =0.2,\upsilon_{m,k}=0.5\right)$ > rate$\left(\varepsilon =0.1,\upsilon_{m,k}=0.5\right)$. The reason is that, where MUs allow the bigger outage probability, the communication quality of MUs means that it is not easy to be interrupted. Therefore, it allows that FUs transmit more power to improve their performance. Moreover, in the case of same outage probability, we can find that the sum rate of FUs with small variance of estimation error is better than that of bigger variance, e.g., $\upsilon_{m,k}=0.2$. Because the bigger error variance means that the estimated channel is not exact, the channel estimation value ${\hat{g}}_{m,k}$ deviates from the true channel gain $g_{m,k}$ seriously.

**Figure 5:**
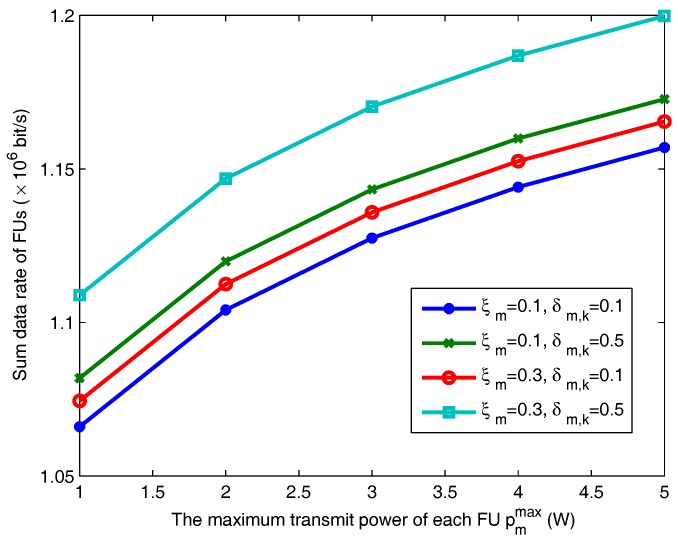
Sum data rate of FUs versus the maximum transmit power under different FU outage probabilities.

Figure 5 depicts the effect of FU’s outage probability $\xi_{m}$ and the variance of channel estimation error ($\Delta h_{m,k}$) on the FU performance. The outage probability and the variance of channel uncertainty of MU are defined as ε=0.1 and $\upsilon_{m,k}=0.01$. The sum data rate of FUs increases with the increasing transmit power level $p_{m}^{max}$. In addition, as the value of $\delta_{m,k}$ increases, i.e., the estimation error increases, the transmission rate of FUs increases accordingly, since it needs more transmit power to overcome the effect of channel uncertainty so that the basic rate requirement of each FU can be satisfied. Additionally, the data rate of FUs increases with the bigger value of $\varepsilon_{m}$.

**Figure 6:**
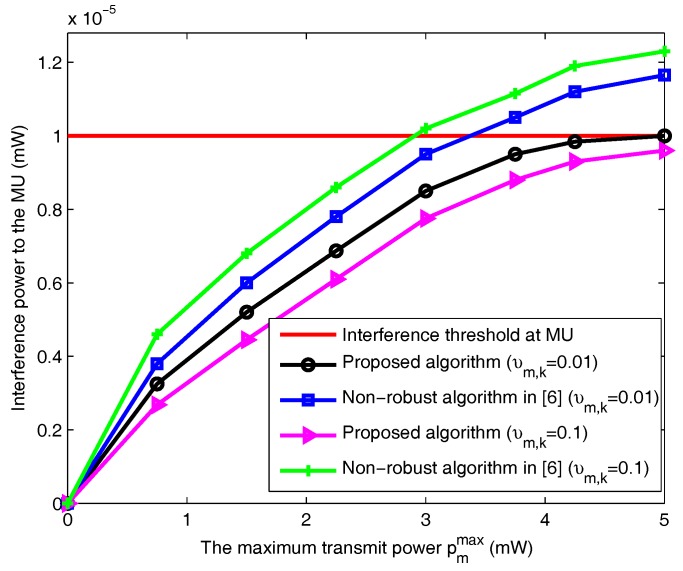
Comparison of interference power to the MU versus maximum transmit power.

Figure 6 shows the interference power to the MU under different channel uncertainties. The outage probability of MU is ε=0.1. The outage probability and variance of FU are defined as $\xi_{m}=0.1$ and $\delta_{m,k}=0.1$ separately. From Figure 6, the interference power to the MU of the proposed algorithm and the non-robust algorithm in [6] increases with the bigger transmit power threshold $p_{m}^{max}$. Because the bigger transmit power can provide more wider feasible region. Additionally, the interference power received by MU in [6] exceeds the threshold $I^{th}$. But the interference to the MU of our method is below the interference power threshold. Additionally, the bigger upper bound of uncertainty $\upsilon_{m,k}$ is, more estimation errors are in the communication system. Therefore, the interference power to MUs under $\upsilon_{m,k}=0.1$ is bigger than the interference power under $\upsilon_{m,k}=0.01$.

**Figure 7:**
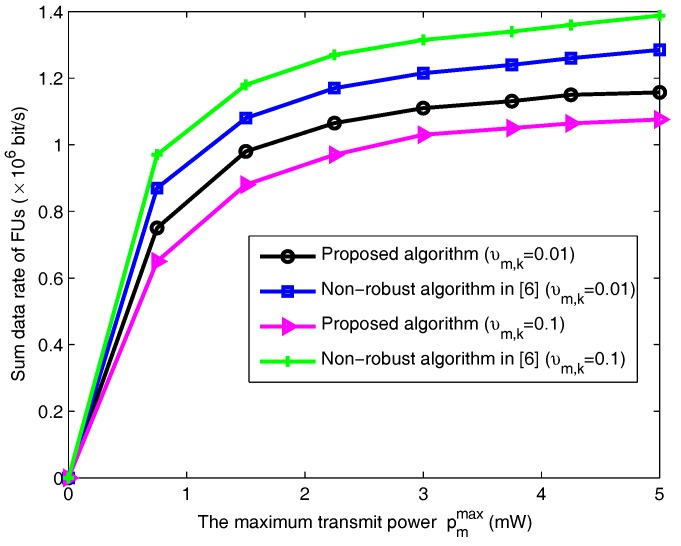
The effect of channel uncertainty on the data rate of FUs.

In Figure 7, the achievable data rate of FUs versus the total power level for the above mentioned schemes is given under different variances of channel estimation errors over FU-to-MU links. From Figure 7, the sum data rate of FUs under the proposed algorithm and the non-robust algorithm increases with the bigger maximum transmit power level. Additionally, as expected, the sum data of FUs under the non-robust algorithm in [6] is higher than our proposed algorithm for a given transmit power upper bound if perfect CSI is assumed, because the allowable transmit power is higher. Moreover, the sum data of FUs converges to an equilibrium point (i.e., saturation state). The reason is that the optimal power is limited by the interference power threshold under the higher transmit power region. In order to protect the QoS of MUs and the limitation of maximum transmit power, it does not allow the FUs to increase their transmission power endlessly.

## 6. Conclusions

In this paper, to well protect the QoS of macrocell cellular networks and improve system capacity, we have formulated a sum rate maximization problem for a two-tier heterogeneous network with one macrocell and multiple femtocells. Due to the effect of channel uncertainties in inter-tier interference channels and the forward channel of femtocell user, we have imposed the robust interference constraint and the robust rate constraint under outage-based uncertainty models to protect the transmission performance of the macrocell and the femtocells. With the scenarios of no CSI feedback and partial CSI feedback, the outage-based probability constraints are transformed into closed-from expressions by approximate methods. With the Lagrange dual method, the robust power allocation problem has been decomposed into a dual problem. The robust power allocation algorithm is proposed by using the sub-gradient method to solve the primal problem. Simulation results have demonstrated that the proposed algorithm can fast converge to the optimal value. The efficient resource allocation with the consideration of channel uncertainties in heterogeneous networks will be investigated in our future work. Green communication emphasizes on incorporating energy awareness in communication systems. Therefore, energy-efficient RA has attracted more attention in both industry and academia recently. In the future, we will focus on the issue of energy efficiency improvement.

## Figures and Tables

**Table 1:** A summary of the related work about RA algorithm of HetNets.

CSI	System Model		Problem Focus		Common Solution Approach	
Perfect	Uplink	[6,7]	Rate maximization	[6,7]	Dual decomposition theory	[6]
					Interference mitigation strategy	[7]
	Downlink	[6,8]	EE maximization	[8]		
					Game strategy	[8]
Imperfect	Worst-case	[12,13,14,15,16]	Rate maximization	[12,13,14,16,20]	MINLP	[12]
					Stackelberg	[13,15,18,19]
			Power minimization	[15]	Lagrangian game theory	[14]
	Stochastic model	[18,19,20,21]			Geometrical programming method	[16]
			Increase social utility	[18,19,21]	Iterative approach	[20]
					Semidefinite programming	[21]
Problem analysis	From the aspect of multiuser network scenario, the robust SINR of FU and robust interference constraint of MU are not considered simultaneously, which can not be ignored.The problem considered in this paper is more comprehensive and complex, more satisfying the heterogeneous network robust transmission scenario.					

**Table 2:** Notation of Symbol.

Symbol	Explanation
$\rho_{m,k}$	subcarrier assignment of FU *m* on subcarrier *k*
$\sigma_{m,k}$	background noise of FU *m* on subcarrier *k*
$p_{m,k}$	transmit power of the *m*th FU over subcarrier *k*
$h_{m,k}$	direct channel gain of FU *m* over subcarrier *k*
$r_{m,k}$	data rate of FU *m* over subcarrier *k*
$I^{th}$	interference temperature level at MU-receiver
$g_{m,k}$	channel gain of FU-to-MU link on subcarrier *k*
$p_{m}^{max}$	maximum transmit power of FU *m*
$R_{m}^{min}$	minimum rate requirement of FU *m*
*B*	bandwidth of each subcarrier

**Table 3:** Simulation parameters.

System Parameters	Values
Number of FUs *M*	4
Number of subcarriers *K*	128
Bandwidth of each subcarrier *B*	10 KHz
The background noise $\sigma_{m,k}$	$1\times 10^{-8}$ mW
Allowable interference level $I^{th}$	$1\times 10^{-5}$ mW
Estimated channel gains ${\hat{g}}_{m,k}$, ${\hat{h}}_{m,k}$	[0,1] [31]
Outage probability threshold of MU ε	[0,1]
Outage probability threshold of FU $\xi_{m}$	[0,1]
Minimum data requirement $R_{m}^{min}$	$2\times 10^{5}$ bit/s
Maximum transmit power $p_{m}^{max}$	5 mW

## Appendix A

According to (1) and (8), we have

(A1) $$\prod_{k=1}^{K}\mathrm{Pr}\left\{B\rho_{m,k}\log_{2}\left(1+\frac{p_{m,k}h_{m,k}}{\sigma_{m,k}^{}}\right)\leq R_{m}^{min}\right\}\leq \xi_{m},$$

and

(A2) $$\prod_{k=1}^{K}\mathrm{Pr}\left\{h_{m,k}\leq \frac{\sigma_{m,k}}{p_{m,k}}\left(2^{R_{m}^{min}/\left(B\rho_{m,k}\right)}-1\right)\right\}\leq \xi_{m}.$$

If we do not know the distribution function of channel gain $h_{m,k}$, similarly, we have the following result

(A3) $$\prod_{k=1}^{K}H_{h_{m,k}}\left(Z_{m,k}\right)\leq \xi_{m},$$

where $Z_{m,k}=\frac{\sigma_{m,k}}{p_{m,k}}\left(2^{R_{m}^{min}/\left(B\rho_{m,k}\right)}-1\right)$. And $H_{h_{m,k}}\left(\cdot \right)$ denotes the CDF of channel $h_{m,k}$. Since we can not directly obtain the relationship between transmit power $p_{m,k}$ and outage probability $\xi_{m}$, therefore, we reform the constraint into the following approximation type, i.e.,

(A4) $$\prod_{k=1}^{K}H_{h_{m,k}}\left(Z_{m,k}\right)\leq KH_{h_{m,\bar{k}}}\left(Z_{m,\bar{k}}\right),$$

where $\bar{k}=\arg\max H_{h_{m,k}}\left(Z_{m,k}\right)$ is a channel selection factor, which is equivalent to

(A5) $$\bar{k}=\arg\min_{\forall k}\frac{h_{m,k}}{\sigma_{m,k}}.$$

In practice, considering that the outage probability requirement of each subcarrier can be satisfied under the worst-case channel environment, the sum of outage set in (A2) can limit the outage probability under the threshold $\xi_{m}$. Therefore, from (A3) to (A5), we obtain the following expression

(A6) $$\begin{array}{c} KH_{h_{m,\bar{k}}}\left(Z_{m,\bar{k}}\right)\overset{\Delta }{\;=}KH_{h_{m,k}}\left(\frac{\sigma_{m,k}}{p_{m,k}}\left(2^{R_{m}^{min}/\left(B\rho_{m,k}\right)}-1\right)\right)\leq \xi_{m}, \end{array}$$

(A7) $$\frac{\sigma_{m,k}\left(2^{R_{m}^{min}/\left(B\rho_{m,k}\right)}-1\right)}{H_{h_{m,k}}^{-1}\left(\xi_{m}/K\right)}\leq p_{m,k}.$$

Therefore, if transmit power of SU over each subcarrier satisfies the constraint (A7), the outage constraint in $C_{7}$ can be ensured. For the sake of simplification, the constraint (A7) also can be expressed as

(A8) $$R_{m}^{min}\leq B\rho_{m,k}\log_{2}\left(1+\frac{p_{m,k}}{\sigma_{m,k}}H_{h_{m,k}}^{-1}\left(\xi_{m}/K\right)\right).$$

The proof is completed.

## Appendix B

To prove the inequality (10), the upper bound of outage-based interference constraint satisfies

(A9) $$\begin{array}{cc} \mathrm{Pr}\left\{\sum_{k=1}^{K}\sum_{m=1}^{M}\rho_{m,k}p_{m,k}g_{m,k}\geq I^{th}\right\} & \leq \mathrm{Pr}\left\{\max_{\forall m,k}\left\{\rho_{m,k}p_{m,k}g_{m,k}\right\}\geq \frac{I^{th}}{K}\right\}\\ & =1-\mathrm{Pr}\left\{\max_{\forall m,k}\left\{\rho_{m,k}p_{m,k}g_{m,k}\right\}\leq \frac{I^{th}}{K}\right\}\\ & =1-\prod_{k=1}^{K}\prod_{m=1}^{M}\mathrm{Pr}\left\{\rho_{m,k}p_{m,k}g_{m,k}\leq \frac{I^{th}}{K}\right\}\\ & =1-{\left[\mathrm{Pr}\left\{\rho_{m,k}p_{m,k}g_{m,k}\leq \frac{I^{th}}{K}\right\}\right]}^{MK}\leq \varepsilon . \end{array}$$

Since the outage probability level ε satisfies the interval of [0,1], we have

(A10) $$\sqrt[{MK}]{1-\varepsilon }\leq \mathrm{Pr}\left\{\rho_{m,k}p_{m,k}g_{m,k}\leq \frac{I^{th}}{K}\right\}.$$

Without the knowledge of PDF of channel gain $g_{m,k}$, we have

(A11) $$\left\{\begin{array}{c} \sqrt[{MK}]{1-\varepsilon }\leq G_{g_{m,k}}\left(\frac{I^{th}}{\rho_{m,k}p_{m,k}K}\right)\\ \rho_{m,k}p_{m,k}\leq \frac{I^{th}}{KG_{g_{m,k}}^{-1}\left(\sqrt[{MK}]{1-\varepsilon }\right)} \end{array}\right.,$$

where $G_{g_{m,k}}\left(\cdot \right)$ and $G_{g_{m,k}}^{-1}\left(\cdot \right)$ denotes the cumulative distribution function (CDF) and the corresponding inverse function of variable $g_{m,k}$, respectively. The proof is completed.

## Appendix C

Based on the formula of Taylor series of the two element function, we have

(A12) $$\begin{array}{c} u^{*}\left({\hat{h}}_{m,k}+\Delta h_{m,k},{\hat{g}}_{m,k}+\Delta g_{m,k}\right)=u^{*}\left({\hat{h}}_{m,k},{\hat{g}}_{m,k}\right)+\sum_{m=1}^{M}\sum_{k\in C_{m}}^{}\frac{\partial u^{*}\left({\hat{h}}_{m,k},{\hat{g}}_{m,k}+\Delta g_{m,k}\right)}{\partial \Delta h_{m,k}}\Delta h_{m,k}+o\left[{\left(\Delta h_{m,k}\right)}^{n}\right]\\ +\sum_{m=1}^{M}\sum_{k\in C_{m}}\frac{\partial u^{*}\left({\hat{h}}_{m,k}+\Delta h_{m,k},{\hat{g}}_{m,k}\right)}{\partial \Delta g_{m,k}}\Delta g_{m,k}+o\left[{\left(\Delta g_{m,k}\right)}^{n}\right],\left(\Delta h_{m,k}\to 0,\Delta g_{m,k}\to 0\right), \end{array}$$

where $o\left[{\left(\Delta g_{m,k}\right)}^{n}\right]$ and $o\left[{\left(\Delta h_{m,k}\right)}^{n}\right]$ denote the corresponding high order infinitesimal small quantities. In addition, $u^{*}\left({\hat{h}}_{m,k},{\hat{g}}_{m,k}\right)$ is the optimal utility function without estimation errors (assuming that the estimated channel gains are equal to the true channel gains).

Ignoring the effect of high order small variables, the sum rate gap between our robust power allocation algorithm and the non-robust algorithm can be written as

(A13) $$\begin{array}{c} U_{gap}=u_{rob}^{*}-u_{non}^{*}=\sum_{m=1}^{M}\sum_{k\in C_{m}}^{}\frac{\partial u^{*}\left({\hat{h}}_{m,k},{\hat{g}}_{m,k}+\Delta g_{m,k}\right)}{\partial \Delta h_{m,k}}\Delta h_{m,k}+\sum_{m=1}^{M}\sum_{k\in C_{m}}\frac{\partial u^{*}\left({\hat{h}}_{m,k}+\Delta h_{m,k},{\hat{g}}_{m,k}\right)}{\partial \Delta g_{m,k}}\Delta g_{m,k}, \end{array}$$

where $u_{rob}^{*}=u^{*}\left({\hat{h}}_{m,k}+\Delta h_{m,k},{\hat{g}}_{m,k}+\Delta g_{m,k}\right)$ is the total data rate with imperfect CSI and $u_{non}^{*}\;=\;u^{*}\left({\hat{h}}_{m,k},{\hat{g}}_{m,k}\right)$ is the total rate with perfect CSI. According to the sensitivity analysis in [32], under the consideration of small uncertainties, we have

(A14) $$\left\{\begin{array}{c} \frac{\partial u^{*}\left({\hat{h}}_{m,k},{\hat{g}}_{m,k}+\Delta g_{m,k}\right)}{\partial \Delta h_{m,k}}\approx -\mu_{m}^{*}\\ \frac{\partial u^{*}\left({\hat{h}}_{m,k}+\Delta h_{m,k},{\hat{g}}_{m,k}\right)}{\partial \Delta g_{m,k}}\approx -\upsilon^{*} \end{array}\right.,$$

According to the relationship (A13) and (A14), we have the following expresssion

(A15) $$\begin{array}{c} U_{gap}=u_{rob}^{*}-u_{non}^{*}=-\sum_{m=1}^{M}\sum_{k\in C_{m}}\left(2\mu_{m}^{*}\delta_{m,k}\xi_{m}+\upsilon^{*}Q^{-1}\left(1-\varepsilon \right)\upsilon_{m,k}\right), \end{array}$$

where $\Delta h_{m,k}=2\delta_{m,k}\xi_{m}$ and $\Delta g_{m,k}=Q^{-1}\left(1-\varepsilon \right)\upsilon_{m,k}$. The proof is completed.
